# Enhancement of drought tolerance in diverse *Vicia faba* cultivars by inoculation with plant growth-promoting rhizobacteria under newly reclaimed soil conditions

**DOI:** 10.1038/s41598-021-02847-2

**Published:** 2021-12-17

**Authors:** Elsayed Mansour, Hany A. M. Mahgoub, Samir A. Mahgoub, El-Sayed E. A. El-Sobky, Mohamed I. Abdul-Hamid, Mohamed M. Kamara, Synan F. AbuQamar, Khaled A. El-Tarabily, El-Sayed M. Desoky

**Affiliations:** 1 Department of Crop Science, Faculty of Agriculture, Zagazig University, Zagazig, 44519 Egypt; 2 Department of Botany and Microbiology, Faculty of Science, Al-Azhar University, Cairo, 11884 Egypt; 3 Agricultural Microbiology Department, Faculty of Agriculture, Zagazig University, Zagazig, 44519 Egypt; 4 Department of Agronomy, Faculty of Agriculture, Kafrelsheikh University, Kafr El-Sheikh, 33516 Egypt; 5 Department of Biology, College of Science, United Arab Emirates University, Al Ain, 15551 United Arab Emirates; 6 Harry Butler Institute, Murdoch University, Murdoch, WA 6150 Australia; 7 Botany Department, Faculty of Agriculture, Zagazig University, Zagazig, 44519 Egypt

**Keywords:** Plant physiology, Drought

## Abstract

Water deficit has devastating impacts on legume production, particularly with the current abrupt climate changes in arid environments. The application of plant growth-promoting rhizobacteria (PGPR) is an effective approach for producing natural nitrogen and attenuating the detrimental effects of drought stress. This study investigated the influence of inoculation with the PGPR *Rhizobium leguminosarum* biovar *viciae* (USDA 2435) and *Pseudomonas putida* (RA MTCC5279) solely or in combination on the physio-biochemical and agronomic traits of five diverse *Vicia faba* cultivars under well-watered (100% crop evapotranspiration [ETc]), moderate drought (75% ETc), and severe drought (50% ETc) conditions in newly reclaimed poor-fertility sandy soil. Drought stress substantially reduced the expression of photosynthetic pigments and water relation parameters. In contrast, antioxidant enzyme activities and osmoprotectants were considerably increased in plants under drought stress compared with those in well-watered plants. These adverse effects of drought stress reduced crop water productivity (CWP) and seed yield‐related traits. However, the application of PGPR, particularly a consortium of both strains, improved these parameters and increased seed yield and CWP. The evaluated cultivars displayed varied tolerance to drought stress: Giza-843 and Giza-716 had the highest tolerance under well-watered and moderate drought conditions, whereas Giza-843 and Sakha-4 were more tolerant under severe drought conditions. Thus, co-inoculation of drought-tolerant cultivars with *R. leguminosarum* and *P. putida* enhanced their tolerance and increased their yield and CWP under water-deficit stress conditions. This study showed for the first time that the combined use of *R. leguminosarum* and *P. putida* is a promising and ecofriendly strategy for increasing drought tolerance in legume crops.

## Introduction

Faba bean (*Vicia faba* L.) is a globally essential pulse crop and is cultivated in the Mediterranean region as a rotational crop^1^. It is a valuable protein source for humans and livestock and plays a major role in improving soil fertility through the symbiotic fixation of atmospheric nitrogen (N), which is critical for low-N environments^2^.

Rainfall fluctuations and shrinking of water bodies are projected to increase, particularly in arid environments, because of abrupt climate change^3,4^. Water deficit is a critical problem that adversely affects the production of field crops worldwide^5,6^. Drought stress has devastating effects on plant metabolism and photosynthesis^7^. Additionally, it leads to increased production of reactive oxygen species (ROS) and oxidation of cellular components, such as proteins, lipids, DNA and RNA^8^. Moreover, ion uptake, nutrient translocation, nutrient assimilation, carbohydrate metabolism, and uptake of growth regulators are disrupted under drought stress^9^.

In general, faba bean is more sensitive to drought than other field crops^10^. Hence, there is a tremendous demand for enhancing its tolerance to drought stress using tolerant genotypes alongside environmentally friendly agronomic approaches^7,11^. The beneficial microbial communities present in soil promote sustainable agriculture of faba bean, particularly under detrimental abiotic stresses^12,13^. The application of these microorganisms as drought protecting agents could be considered a promising solution for producing high crop yields under drought stress^14,15^. Inoculating plants with plant growth-promoting rhizobacteria (PGPR) confers more tolerance to the negative effects of environmental stresses, such as salinity, drought, flooding, and heavy metal accumulation^16,17^. PGPR can stimulate plant growth under drought stress by producing indole-3-acetic acid and 1-aminocyclopropane-1-carboxylic acid (ACC)^18,19^.

N is an essential nutrient for plant growth; however, it is deficient in newly reclaimed sandy soils^20,21^. Large-scale N application leads to considerable environmental pollution and has adverse effects on human health^22^. Recently, its price has increased, particularly in developing countries. In contrast, the genus *Rhizobium* generates nodules on the roots of their hosts and fixes a large amount of N in a symbiotic relationship in legume crops^23^.

Although many studies have investigated the inoculation of legume crops with PGPR, additional investigation is needed to explore the physio-biochemical and agronomic responses of faba bean genotypes to PGPR application under varying irrigation regimes in poor soils in arid regions. We hypothesized that the application of PGPR could remarkably enhance the physio-biochemical, morphological, and agronomic traits of faba bean under drought stress in arid environments. The objective of this study was to evaluate the effects of inoculation with *Rhizobium leguminosarum* and/or *Pseudomonas putida* on faba bean in newly reclaimed poor-fertility sandy soil under varying irrigation regimes in arid environments at the physiological, biochemical, morphological, and agronomic levels. Furthermore, we evaluated recommended commercial cultivars to identify drought-tolerant high-yielding faba bean genotypes that could be cultivated in arid Mediterranean environments.

## Results

### Chlorophyll, proline, and soluble sugar contents

**Figure 1:**
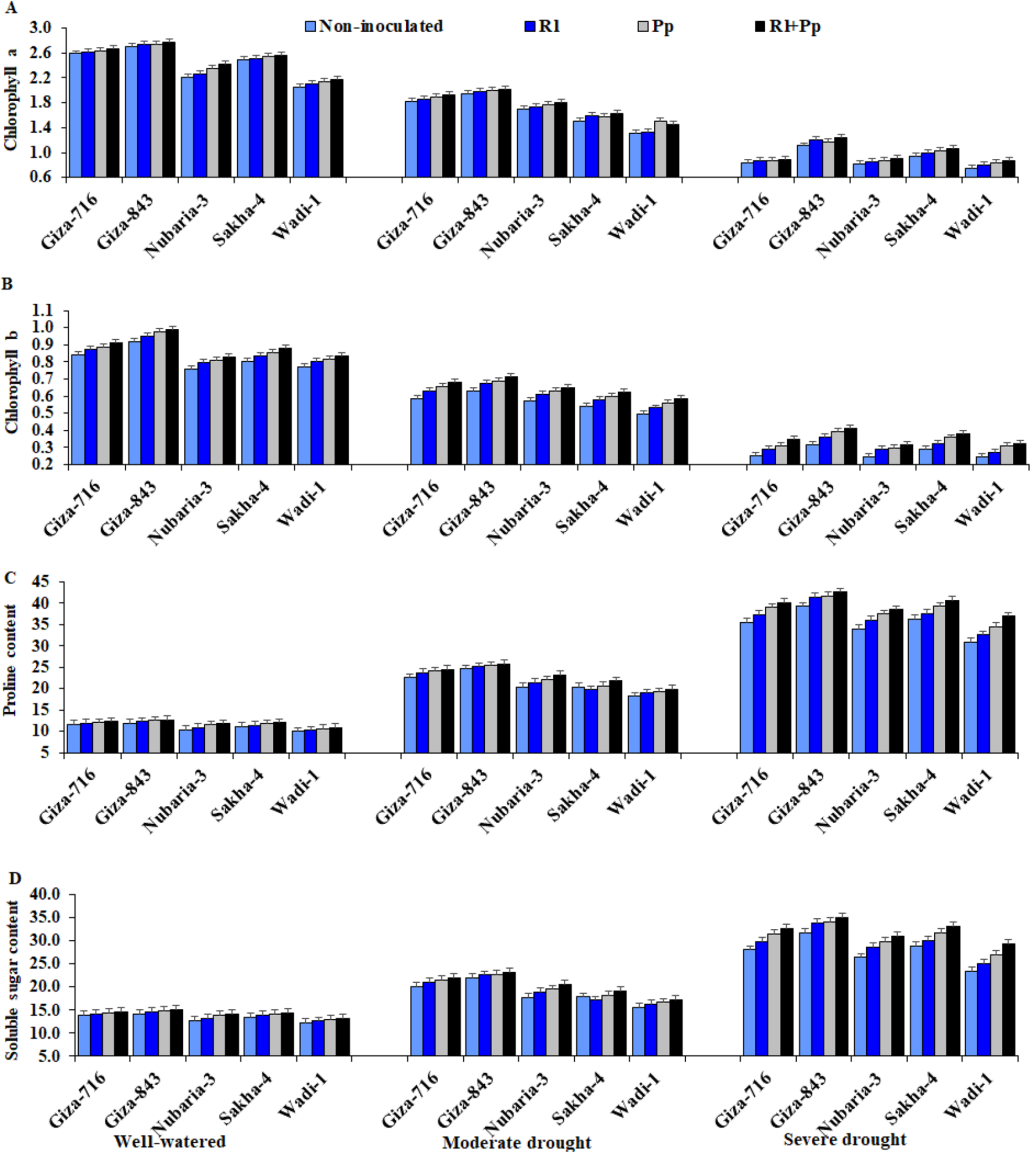
Effect of inoculation with *Rhizobium leguminosarum* (Rl) and *Pseudomonas putida* (Pp) on the chlorophyll *a* level (**A**), chlorophyll *b* level (**B**), proline content (**C**), and soluble sugar content (**D**) of five faba bean cultivars compared with those in non-inoculated plants (Non) under three irrigation regimes over two growing seasons. The bars on the top of the columns correspond to LSD (*p* ≤ 0.05).

Drought stress had detrimental effects on the levels of chlorophyll *a* (Chl *a*) and *b* (Chl *b*), which steeply decreased, particularly under severe drought conditions (Table 1 and Fig. 1). Chl *a* levels decreased by 30.4% and 61.7% under moderate and severe drought conditions, respectively, compared with those under well-watered conditions. Similarly, Chl *b* levels decreased under moderate and severe drought conditions by 28.7% and 63.2%, respectively, compared with those under well-watered conditions.

**Table 1:** Effect of three irrigation regimes and inoculation with *Rhizobium leguminosarum* (Rl) and *Pseudomonas putida* (Pp) compared with control without inoculation on chlorophyll *a* (Chl *a*, mg g^−1^ FW), chlorophyll *b* (Chl *b*, mg g^−1^ FW), proline content (μmol g^−1^ DW), soluble sugar content (SSC; mg g^−1^ DW), relative water content (RWC, %), membrane stability index (MSI, %), excised leaf water retention (ELWR, %), and relative water loss (RWL, %) of five faba bean cultivars over two growing seasons (2018–2019 and 2019–2020).

Studied factors		Chl *a*	Chl *b*	Proline	SSC	RWC	MSI	ELWR	RWL
**Irrigation**									
Well-watered		2.463^a^	0.856^a^	11.46^c^	13.83^c^	59.71^a^	56.35^a^	73.31^a^	86.07^a^
Moderate drought		1.716^b^	0.611^b^	22.06^b^	19.48^b^	46.99^b^	43.01^b^	60.59^b^	73.35^b^
Severe drought		0.944^c^	0.316^c^	37.58^a^	30.05^a^	34.38^c^	30.39^c^	47.98^c^	60.74^c^
**Cultivar**									
Giza-716		1.833^b^	0.617^b^	24.69^b^	22.11^b^	48.61^b^	44.84^b^	62.21^b^	74.97^b^
Giza-843		1.966^a^	0.668^a^	26.26^a^	23.68^a^	50.37^a^	46.60^a^	63.97^a^	76.73^a^
Nubaria-3		1.641^c^	0.566^d^	23.11^c^	20.53^c^	46.11^c^	42.33^c^	59.71^c^	72.47^c^
Sakha-4		1.656^c^	0.576^c^	23.41^c^	20.83^c^	45.98^c^	42.20^c^	59.58^c^	72.34^c^
Wadi-1		1.441^d^	0.545^e^	21.04^d^	18.46^d^	44.05^d^	40.28^d^	57.65^d^	70.41^d^
**Bacteria**									
Without inoculation		1.649^d^	0.550^d^	22.45^d^	19.87^d^	44.48^d^	40.70^d^	58.08^d^	70.84^d^
*Rhizobium leguminosarum* (Rl)		1.696^c^	0.587^c^	23.37^c^	20.79^c^	46.64^c^	42.86^c^	60.24^c^	73.00^c^
*Pseudomonas putida* (Pp)		1.726^b^	0.609^b^	24.10^b^	21.52^b^	47.86^b^	44.08^b^	61.46^b^	74.22^b^
Rl + Pp		1.758^a^	0.631^a^	24.89^a^	22.31^a^	49.12^a^	45.34^a^	62.72^a^	75.48^a^

ANOVA	DF	*p*-value							
Irrigation (I)	2	< 0.001	< 0.001	< 0.001	< 0.001	< 0.001	< 0.001	< 0.001	< 0.001
Cultivar (C)	4	< 0.001	< 0.001	< 0.001	< 0.001	< 0.001	< 0.001	< 0.001	< 0.001
Bacteria (B)	3	< 0.001	< 0.001	< 0.001	< 0.001	< 0.001	< 0.001	< 0.001	< 0.001
I × C	8	< 0.001	< 0.001	< 0.001	< 0.001	0.001	0.001	0.001	0.001
I × B	6	0.023	0.052	0.002	0.001	0.150	0.148	0.152	0.150
B × C	12	< 0.001	0.038	0.025	0.025	0.013	0.025	0.033	0.035
I × B × C	24	0.003	0.045	0.034	0.043	0.021	0.048	0.036	0.0419

Means followed by different letters under the same factor differ significantly by LSD at *p* ≤ 0.05.

Notwithstanding, *R. leguminosarum* and *P. putida* inoculants and their combination significantly increased Chl *a* and Chl *b* levels in all faba bean cultivars under the three irrigation regimes. The combination treatment recorded the highest Chl *a* and Chl *b* levels, followed by inoculation with *R. leguminosarum* and *P. putida* alone*.* The Chl *a* and Chl *b* levels increased by 6.6% and 14.7%, respectively, following combination treatment compared with those in non-inoculated plants.

The Giza-843 and Giza-716 cultivars displayed the highest Chl *a* and Chl *b* levels under well-watered and moderate drought conditions, whereas the Giza-843 and Sakha-4 cultivars displayed the highest values under severe drought conditions (Fig. 1). Moreover, the Giza-843 and Sakha-4 cultivars inoculated with both strains displayed the highest Chl *a* and Chl *b* levels under severe drought conditions. In contrast, Wadi-1 recorded the lowest values under both drought conditions.

Drought stress significantly increased the proline and soluble sugar contents, particularly under severe drought conditions compared with that under well-watered conditions. The proline content increased by 92.6% and 228% and soluble sugar content increased by 40.9% and 117.3% under moderate and severe drought conditions, respectively, compared with those under well-watered conditions. Inoculation with *R. leguminosarum* and *P. putida* and their combination significantly increased the proline and soluble sugar contents in all evaluated cultivars under the three irrigation regimes compared with those in the non-inoculated plants (Fig. 1).

Co-inoculation with both strains increased the proline and soluble sugar contents by 10.9% and 12.3%, respectively, compared with those in the non-inoculated plants. The Giza-843, Giza-716, and Sakha-4 cultivars inoculated with both strains exhibited the highest proline and soluble sugar contents under moderate and severe drought conditions (Fig. 1). In contrast, Wadi-1 showed the lowest proline and soluble sugar contents under water-deficit conditions.

### Water relations

**Figure 2:**
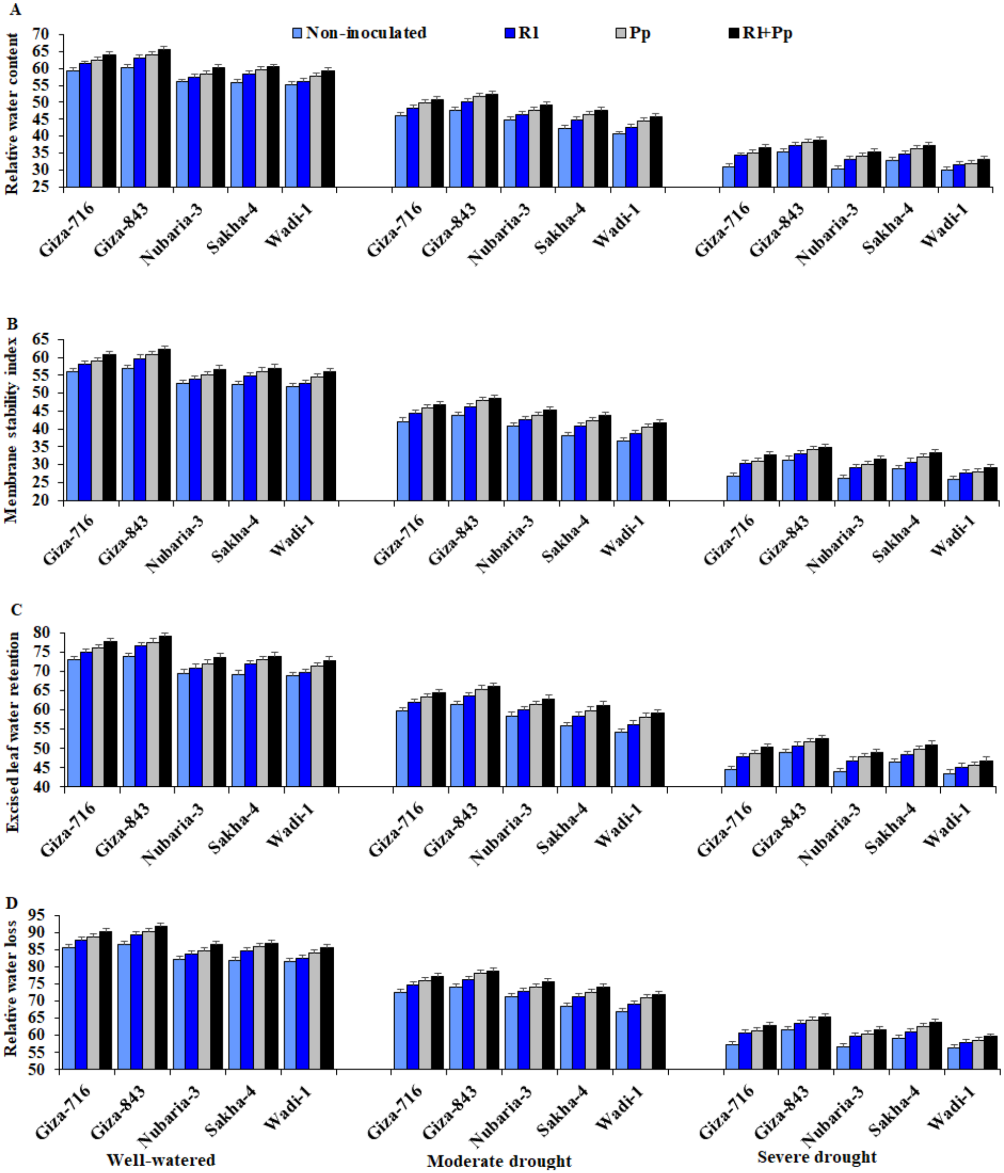
Effect of inoculation with *Rhizobium leguminosarum* (Rl) and *Pseudomonas putida* (Pp) on the relative water content (**A**), membrane stability index (**B**), excised leaf water retention (**C**), and relative water loss (**D**) of five faba bean cultivars compared with those in non-inoculated plants (Non) under three irrigation regimes over two growing seasons. The bars on the top of the columns correspond to LSD (*p* ≤ 0.05).

The relative water content (RWC), membrane stability index (MSI), relative water loss (RWL), and excised leaf water retention (ELWR) significantly decreased because of drought stress in all evaluated faba bean cultivars (Table 1 and Fig. 2). Severe drought stress decreased RWC, MSI, RWL, and ELWR by 42.4%, 46.1%, 29.4%, and 34.6%, respectively, compared with those in well-watered conditions.

In contrast, plants inoculated with *R. leguminosarum* and/or *P. putida* had significantly higher RWC, MSI, ELWR, and RWL than non-inoculated plants under the three irrigation regimes. Inoculation with a combination of both strains resulted in maximum values of these parameters, followed by treatment with either *P. putida* or *R. leguminosarum* (Table 1). The combination of both strains increased RWC, MSI, RWL, and ELWR by 10.4%, 11.4%, 6.6%, and 8.0%, respectively, compared with those in the non-inoculated plants. The Giza-843 and Sakha-4 cultivars inoculated with both strains exhibited the highest RWC, MSI, ELWR, and RWL values under severe drought conditions (Fig. 2). On the other hand, the lowest values were recorded for Wadi-1 under both drought stress conditions.

### Enzymatic and non-enzymatic antioxidant activities

**Figure 3:**
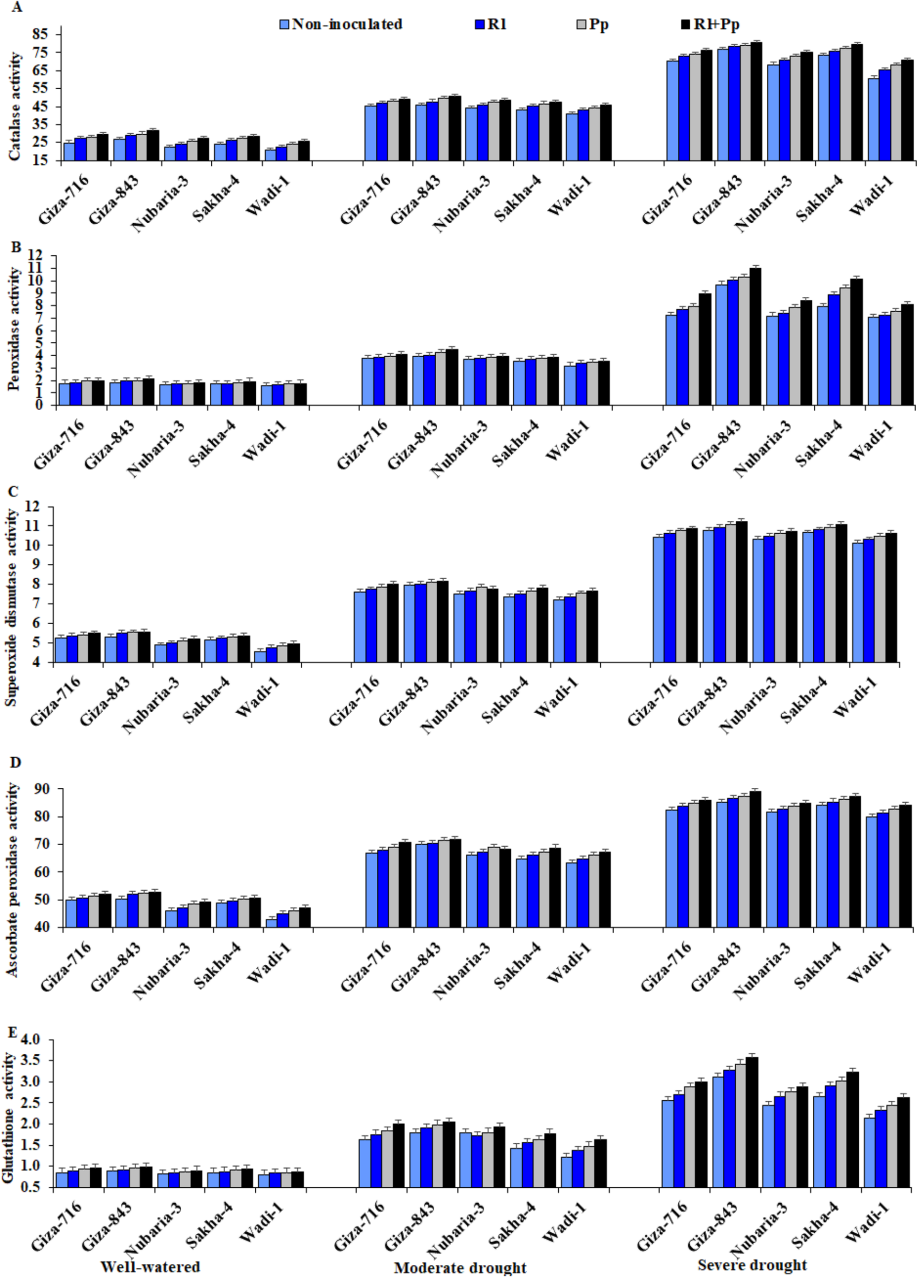
Effect of inoculation with *Rhizobium leguminosarum* (Rl) and *Pseudomonas putida* (Pp) on the catalase (**A**), peroxidase (**B**), superoxide dismutase (**C**), ascorbate peroxidase (**D**), and glutathione (**E**) activities of five faba bean cultivars compared with those in non-inoculated plants (Non) under three irrigation regimes over two growing seasons. The bars on the top of the columns correspond to LSD (*p* ≤ 0.05).

The activities of antioxidant enzymes, including catalase (CAT), peroxidase (POX), superoxide dismutase (SOD), and ascorbate peroxidase (APX), and non-enzymatic antioxidants, including glutathione (GSH) and α-tocopherol (α-Toc), were significantly affected by irrigation regimes, faba bean cultivars, bacterial inoculations, and their interactions (Table 2 and Fig. 3).

**Table 2:** Effects of three irrigation regimes and inoculation with *Rhizobium leguminosarum* (Rl) and *Pseudomonas putida* (Pp) compared with control without inoculation on the activities of catalase (CAT, unit mg^−1^ protein), peroxidase (POX, unit mg^−1^ protein), superoxide dismutase (SOD, unit mg^−1^ protein), ascorbate peroxidase (APX, unit mg^−1^ protein), glutathione (GSH, µmol g^−1^ FW), and α-tocopherol (α-TOC, µmol g^−1^ DW) of five faba bean cultivars over two growing seasons (2018–2019 and 2019–2020).

Studied factors			CAT	POX	SOD	APX	GsH	α-TOC
**Irrigation**								
Well-watered			26.22^c^	1.81^c^	5.17^c^	49.15^c^	0.882^c^	1.578^c^
Moderate drought			46.28^b^	3.80^b^	7.71^b^	67.86^b^	1.711^b^	2.801^b^
Severe drought			73.38^a^	8.50^a^	10.69^a^	84.48^a^	2.825^a^	4.241^a^
**Cultivar**								
Giza-716			50.49^b^	4.96^b^	8.01^a^	68.49^b^	1.883^b^	2.990^b^
Giza-843			52.15^a^	5.45^a^	8.18^a^	69.93^a^	2.068^a^	3.261^a^
Nubaria-3			47.74^d^	4.42^c^	7.75^b^	66.20^d^	1.781^c^	2.799^c^
Sakha-4			48.50^c^	4.49^c^	7.83^b^	66.94^c^	1.753^c^	2.822^c^
Wadi-1			44.27^e^	4.19^d^	7.53^c^	64.25^e^	1.544^d^	2.494^d^
**Bacteria**								
Without inoculation			45.88^d^	4.39^d^	7.67^d^	65.51^d^	1.660^d^	2.634^d^
*Rhizobium leguminosarum* (Rl)			48.04^c^	4.59^c^	7.81^c^	66.73^c^	1.766^c^	2.814^c^
*Pseudomonas putida* (Pp)			49.49^b^	4.77^b^	7.93^b^	67.78^b^	1.847^b^	2.945^b^
Rl + Pp			51.10^a^	5.07^a^	8.03^a^	68.63^a^	1.951^a^	3.100^a^

ANOVA		DF	*p*-value					
Irrigation (I)		2	< 0.001	< 0.001	< 0.001	< 0.001	< 0.001	< 0.001
Cultivar (C)		4	< 0.001	< 0.001	< 0.001	< 0.001	< 0.001	< 0.001
Bacteria (B)		3	< 0.001	< 0.001	< 0.001	< 0.001	< 0.001	< 0.001
I × C		8	< 0.001	< 0.001	< 0.001	< 0.001	< 0.001	< 0.001
I × B		6	0.011	0.041	0.031	0.067	0.024	0.031
B × C		12	< 0.001	< 0.001	0.025	0.021	0.042	0.045
I × B × C		24	< 0.001	< 0.001	0.040	0.076	0.019	0.021

Means followed by different letters under the same factor differ significantly by LSD at *p* ≤ 0.05.

CAT, POX, SOD, APX, GSH, and α-Toc activities were significantly increased in all evaluated cultivars in drought stress conditions compared with those in well-watered conditions. Severe drought stress significantly increased CAT, POX, SOD, APX, GSH, and α-Toc activities by 179%, 268%, 106%, 72%, 220%, and 168%, respectively, compared with that in well-watered conditions (Table 2). In contrast, treatment with *R. leguminosarum* and/or *P. putida* increased the CAT, POX, SOD, APX, GSH, and α-Toc activities in all cultivars under drought stress.

The most effective application was the combination of both strains, which increased the CAT, POX, SOD, APX, GSH, and α-Toc activities by 11.4%, 15.5%, 4.7%, 4.8%, 17.5% and 17.7%, respectively, compared with those in non-inoculated plants (Table 2). The cultivars showed diverse enzymatic and non-enzymatic antioxidant activities under different irrigation regimes. The Giza-843 and Sakha-4 cultivars inoculated with both bacterial strains showed the highest antioxidant activities under severe drought stress. In contrast, Wadi-1 showed the lowest antioxidant activities under moderate and severe drought stress conditions (Fig. 3).

### Root parameters

**Figure 4:**
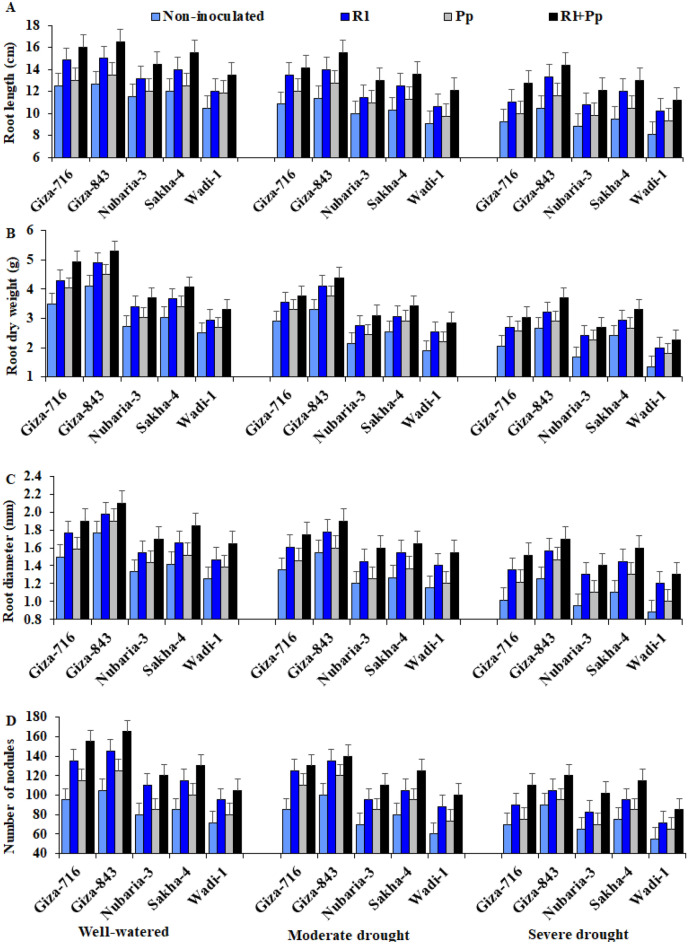
Effect of inoculation with *Rhizobium leguminosarum* (Rl) and *Pseudomonas putida* (Pp) on the root length (**A**), root dry weight (**B**), root diameter (**C**) and number of nodules (**D**) of five faba bean cultivars compared with those in non-inoculated plants (Non) under three irrigation regimes over two growing seasons. The bars on the top of the columns correspond to LSD (*p* ≤ 0.05).

The evaluated root parameters, i.e., root length, root dry weight (DW), root diameter, and number of nodules, were also significantly affected by irrigation regimes, cultivars, bacterial inoculations, and their interactions (Table 3 and Fig. 4). Drought stress had a negative effect on all tested root parameters compared with that in well-watered conditions. The effect was more pronounced under severe drought conditions (50% crop evapotranspiration [ETc]) than under moderate drought conditions (75% ETc).

**Table 3:** Effect of three irrigation regimes and inoculation with *Rhizobium leguminosarum* (Rl) and *Pseudomonas putida* (Pp) compared with control without inoculation on root length (cm), root dry weight (g), root diameter (mm), and number of nodules of five faba bean cultivars over two growing seasons (2018–2019 and 2019–2020).

Studied Factors		Root length	Root dry weight	Root diameter	Number of nodules
**Irrigation**					
Well-watered		13.35^a^	3.702^a^	1.634^a^	110.8^a^
Moderate drought		11.94^b^	3.046^b^	1.482^b^	101.6^b^
Severe drought		10.91^c^	2.526^c^	1.283^c^	86.03^c^
**Cultivar**					
Giza-716		12.65^b^	3.465^b^	1.531^b^	110.0^b^
Giza-843		13.43^a^	3.903^a^	1.713^a^	120.4^a^
Nubaria-3		11.50^c^	2.690^d^	1.355^d^	89.50^d^
Sakha-4		12.06^bc^	3.039^c^	1.448^c^	98.33^c^
Wadi-1		10.69^d^	2.358^e^	1.286^e^	79.14^e^
**Bacteria**					
Without inoculation		10.46^d^	2.587^d^	1.266^d^	79.89^d^
*Rhizobium leguminosarum* (Rl)		12.56^b^	3.231^b^	1.538^b^	106.1^b^
*Pseudomonas putida* (Pp)		11.39^c^	2.961^c^	1.385^c^	91.89^c^
Rl + Pp		13.85^a^	3.586^a^	1.678^a^	120.8^a^

ANOVA	DF	*p*-value			
Irrigation (I)	2	0.003	< 0.001	< 0.001	< 0.001
Cultivar (C)	4	< 0.001	< 0.001	0.001	< 0.001
Bacteria (B)	3	< 0.001	< 0.001	< 0.001	< 0.001
I × C	8	0.045	0.018	0.018	0.001
I × B	6	0.052	0.047	0.024	< 0.001
B × C	12	0.055	0.021	0.016	0.033
I × B × C	24	0.041	0.077	0.039	0.046

Means followed by different letters under the same factor differ significantly by LSD at *p* ≤ 0.05.

There was a significant reduction in root length, root DW, root diameter, and number of nodules by 18.3%, 31.8%, 21.4%, and 23.0%, respectively, under severe drought conditions compared with those under well-watered conditions (Table 3). The Giza-843, Giza-716, and Sakha-4 cultivars showed the highest values under moderate and severe drought conditions (Fig. 4). In contrast, Wadi-1 was most affected by these two drought conditions. Inoculation with *R. leguminosarum* and *P. putida* either separately or in combination significantly enhanced the root parameters of the five cultivars under the three irrigation regimes compared with those in non-inoculated plants. The improvement in root parameters was more pronounced with the combination treatment, particularly under drought stress conditions. Inoculation with both strains improved the root length, root DW, root diameter, and number of nodules by 32.4%, 38.6%, 32.5% and 51.2%, respectively, compared with those in non-inoculated plants under severe drought conditions (Table 3). The interaction effects showed that the combination treatment presented the highest root parameters in the Giza-843, Giza-716, and Sakha-4 cultivars under drought stress conditions (Fig. 4).

### Yield and yield-related traits

**Figure 5:**
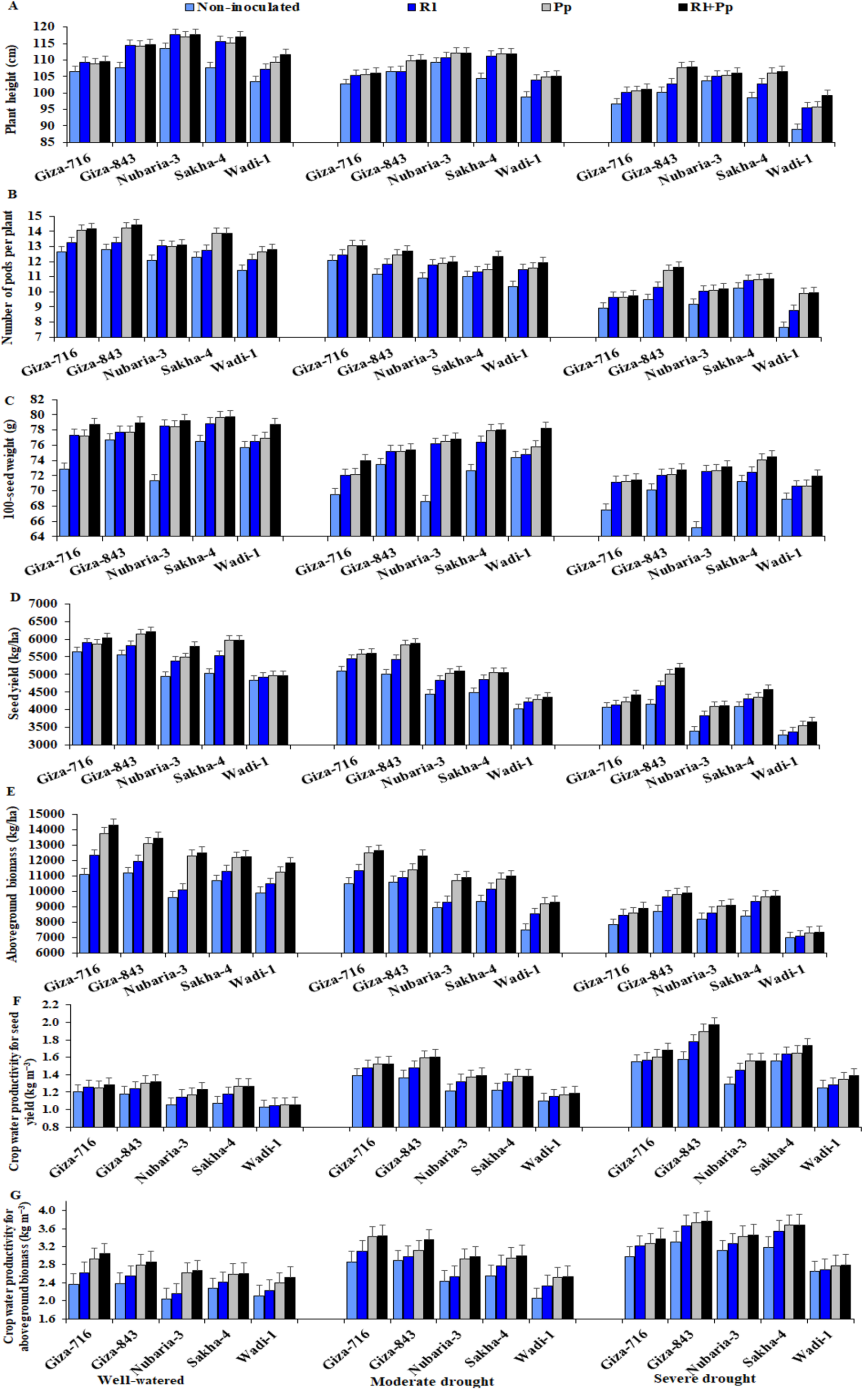
Effect of inoculation with *Rhizobium leguminosarum* (Rl) and *Pseudomonas putida* (Pp) on the plant height (**A**), number of pods per plant (**B**), 100-seed weight (**C**), seed yield (**D**), aboveground biomass (**E**), crop water productivity for seed yield (**F**), and aboveground biomass (**G**) of five faba bean cultivars compared with those in non-inoculated plants (Non) under three irrigation regimes over two growing seasons. The bars on the top of the columns correspond to LSD (*p* ≤ 0.05).

The crop yield and its attributes were gradually and significantly reduced by increasing the drought level in all the cultivars (Table 4 and Fig. 5). Severe drought stress decreased the plant height, number of pods per plant, 100-seed weight, seed yield, and aboveground biomass by 9.3%, 23.9%, 7.8%, 25.7%, and 26.8%, respectively, compared with those under well-watered conditions. *R. leguminosarum* and *P. putida* and their combination exerted stimulatory effects on yield traits in both drought conditions. Compared with non-inoculation, inoculation mitigated the inhibitory effects of water deficit and induced stimulatory effects.

**Table 4:** Effect of three irrigation regimes and inoculation with *Rhizobium leguminosarum* (Rl) and *Pseudomonas putida* (Pp) compared with control without inoculation on plant height (PH, cm), number of pods per plant (NP/P), 100-seed weight (100-SW, g), seed yield (SY, kg ha^−1^), aboveground biomass (AB, kg ha^−1^), and crop water productivity (kg m^−3^) for seed yield (CWP_s_) and aboveground biomass (CWP_ab_) of five faba bean cultivars over two growing seasons (2018–2019 and 2019–2020).

Studied Factors			PH	NP/P	100-SW	SY	AB	CWP_s_	CWP_ab_
**Irrigation**									
Well-watered			111.9^a^	13.09^a^	77.35^a^	5546^a^	11,787^a^	1.179^c^	2.506^c^
Moderate drought			107.4^b^	11.84^b^	74.65^b^	4981^b^	10,402^b^	1.358^b^	2.836^b^
Severe drought			101.5^c^	9.96^c^	71.29^c^	4125^c^	8629^c^	1.567^a^	3.277^a^
**Cultivar**									
Giza-716			104.3^c^	11.89^b^	72.92^d^	5168^b^	11,029^a^	1.448^a^	3.019^a^
Giza-843			108.5^b^	12.13^a^	74.77^bc^	5408^a^	11,086^a^	1.497^a^	3.096^a^
Nubaria-3			110.8^a^	11.44^c^	74.08^c^	4702^d^	9947^c^	1.328^b^	2.835^b^
Sakha-4			109.0^b^	11.79^b^	75.98^a^	4939^c^	10,407^b^	1.339^b^	2.883^b^
Wadi-1			102.0^d^	10.88^d^	74.41^b^	4203^e^	8896^d^	1.215^c^	2.530^c^
**Bacteria**									
Without inoculation			103.2^c^	10.82^c^	71.63^c^	4538^c^	9300^d^	1.269^d^	2.612^d^
*Rhizobium leguminosarum* (Rl)			107.2^b^	11.52^b^	74.80^b^	4842^b^	9978^c^	1.355^c^	2.804^c^
*Pseudomonas putida* (Pp)			108.2^ab^	12.00^a^	75.21^b^	5028^a^	10,781^b^	1.409^b^	3.006^b^
Rl + Pp			109.1^a^	12.18^a^	76.09^a^	5129^a^	11,033^a^	1.439^a^	3.071^a^

ANOVA		DF	*p*-value						
Irrigation (I)		2	< 0.001	< 0.001	< 0.001	< 0.001	< 0.001	< 0.001	< 0.001
Cultivar (C)		4	< 0.001	< 0.001	< 0.001	< 0.001	< 0.001	< 0.001	< 0.001
Bacteria (B)		3	0.001	< 0.001	< 0.001	< 0.001	< 0.001	< 0.001	< 0.001
I × C		8	< 0.001	< 0.001	< 0.001	< 0.001	< 0.001	< 0.001	< 0.001
I × B		6	< 0.001	< 0.001	< 0.001	< 0.001	< 0.001	< 0.001	< 0.001
B × C		12	< 0.001	< 0.001	< 0.001	< 0.001	< 0.001	< 0.001	< 0.001
I × B × C		24	< 0.001	< 0.001	< 0.001	< 0.001	< 0.001	< 0.001	< 0.001

Means followed by different letters under the same factor differ significantly by LSD at *p* ≤ 0.05.

The application that most affected the yield traits was the combination treatment in all the cultivars. Plants inoculated with both strains recorded significant increases in plant height, number of pods per plant, 100-seed weight, seed yield, and aboveground biomass by 5.7%, 12.6%, 6.2%, 13.0% and 18.6%, respectively, compared with those in non-inoculated plants under severe drought conditions (Table 4).

The Giza-843 and Giza-716 cultivars showed the highest seed yield and yield-related traits under moderate drought conditions, whereas the Giza-843 and Sakha-4 cultivars showed the highest values under severe drought conditions, particularly those inoculated with the combination treatment (Fig. 5).

### Crop water productivity (CWP)

The faba bean plants under moderate and severe drought stress conditions exhibited higher CWP for seed yield (CWP_s_) and aboveground biomass (CWP_ab_) than the plants under well-watered conditions (Table 4 and Fig. 5). Drought-stressed plants had higher CWP than well-watered plants because of more efficient water consumption and water loss reduction by the plants as a result of osmotic regulation.

Inoculation with *R. leguminosarum* and *P. putida* significantly improved CWP_s_ and CWP_ab_ compared with those in non-inoculated plants. The highest CWP_s_ and CWP_ab_ were observed in the Giza-843 and Giza-716 cultivars inoculated with both strains under well-watered and moderate drought stress conditions, whereas the Giza-843 and Sakha-4 cultivars inoculated with both strains showed the highest CWP_s_ and CWP_ab_ under severe drought stress (Fig. 5). The lowest CWP_s_ and CWP_ab_ were recorded in the Wadi-1 cultivar under the three irrigation regimes.

### Seed protein pattern determined using sodium dodecyl sulfate–polyacrylamide gel electrophoresis (SDS–PAGE)

The protein banding pattern was examined in the cultivars inoculated with *R. leguminosarum* and *P. putida* individually or in combination. The highest differences in the physiological, biochemical, and agronomic results were observed between the well-watered and severe drought conditions and between the Giza-843 and Wadi-1 cultivars. Accordingly, total seed storage proteins were subjected to SDS–PAGE for the Giza-843 and Wadi-1 cultivars inoculated with *R. leguminosarum* and/or *P. putida* under well-watered and severe drought conditions. Using one-dimensional SDS–PAGE analysis, optical differences in the protein patterns were observed between the two cultivars (Table 5 and Fig. S1).

**Table 5:** Effect of co-inoculation with *Rhizobium leguminosarum* (*Rl*) and/or *Pseudomonas putida (Pp)* on sodium dodecyl sulfate–polyacrylamide gel electrophoresis (SDS–PAGE) seed protein patterns of two *Vicia faba* cultivars *(*Giza-843 and Wadi-1) under well-watered and severe drought conditions compared with that in non-inoculated plants (Non).

Molecular weight (KD)	Under well-watered conditions				Under severe drought conditions			
	1	2	3	4	5	6	7	8
	Non	*Pp*	*Rl*	*Pp* + *Rl*	Non	*Pp*	*Rl*	*Pp* + *Rl*
**Giza-843**								
95	1	1	1	1	1	1	1	1
70	1	1	1	1	1	1	1	1
60	1	1	1	1	1	1	1	1
55	0	0	1	1	1	1	1	1
40	1	1	1	1	1	1	1	1
32	1	1	1	1	1	1	1	1
27	0	0	1	1	1	0	1	1
22	1	1	1	1	1	1	1	1
15	1	1	1	1	1	1	1	1
10	1	1	1	1	1	1	1	1
4	0	0	0	0	0	1	1	1
2	1	1	0	1	1	1	1	1
Total bands	9	9	10	11	11	11	12	12
New bands	0	0	1	2	0	0	1	0

Molecular weight (KD)	Under well-watered conditions				Under severe drought conditions			
	9	10	11	12	13	14	15	16
	Non	*Pp*	*Rl*	*Pp* + *Rl*	Non	*Pp*	*Rl*	*Pp* + *Rl*
**Wadi-1**								
250	1	1	1	1	1	1	1	1
150	1	1	1	1	1	0	0	0
102	1	1	1	1	1	0	0	0
100	1	1	1	1	1	1	1	1
70	1	1	1	1	1	1	1	1
60	1	1	1	1	1	1	1	1
50	1	1	1	1	1	1	1	1
30	1	1	1	1	1	1	1	1
25	1	1	1	1	1	1	1	1
20	1	1	1	1	1	1	1	1
17	1	1	1	1	1	1	1	1
15	1	1	1	1	1	1	1	1
Total bands	12	12	12	12	12	10	10	10
New bands	0	0	0	0	0	0	0	0

1: presence of band(s), 0: absence of band(s).

The molecular weights of the protein subunits varied between 2 and 250 kDa in both cultivars, and the proteins were resolved into 12 bands. Protein bands with molecular weights of 95, 55, 40, 32, 27, 22, 10, 4, and 2 kDa were detected in Giza-843 but not in Wadi-1. In contrast, certain protein bands with molecular weights of 250, 102, 100, 50, 30, 25, 20, and 17 kDa were detected in Wadi-1 but not in Giza-843 (Table 5 and Fig. S1). Nine protein bands with molecular weights of 95, 70, 60, 40, 32, 22, 15, 10, and 2 kDa were detected in the non-inoculated Giza-843 plants under well-watered conditions, whereas 11 protein bands with molecular weights of 95, 70, 60, 55, 40, 32, 27, 22, 15, 10, and 2 kDa were detected in the non-inoculated Giza-843 plants under drought conditions. Hence, two new protein bands with molecular weights of 55 and 27 kDa were identified in non-inoculated plants under drought stress, but these bands were absent in non-inoculated plants under well-watered conditions (Table 5). Moreover, a new band (4 kDa) was detected in plants inoculated with *R. leguminosarum* and *P. putida* under severe drought stress but was absent under well-watered conditions (Table 5).

Twelve protein bands with molecular weights of 250, 150, 102, 100, 70, 60, 50, 30, 25, 20, 17, and 15 kDa were detected in Wadi-1. No differences in protein banding patterns were noted between non-inoculated Wadi-1 plants under well-watered conditions and those under drought conditions (Table 5). In contrast, two protein bands with molecular weights of 150 and 102 kDa were absent in plants inoculated with *R. leguminosarum* and/or *P. putida* under drought conditions. The band intensity increased at molecular weights of 17, 30, and 50 kDa in Wadi-1 compared with that in Giza-843, whereas the band intensity increased at molecular weights of 22 and 40 kDa in Giza-843.

### Association among the evaluated parameters and treatments

**Figure 6:**
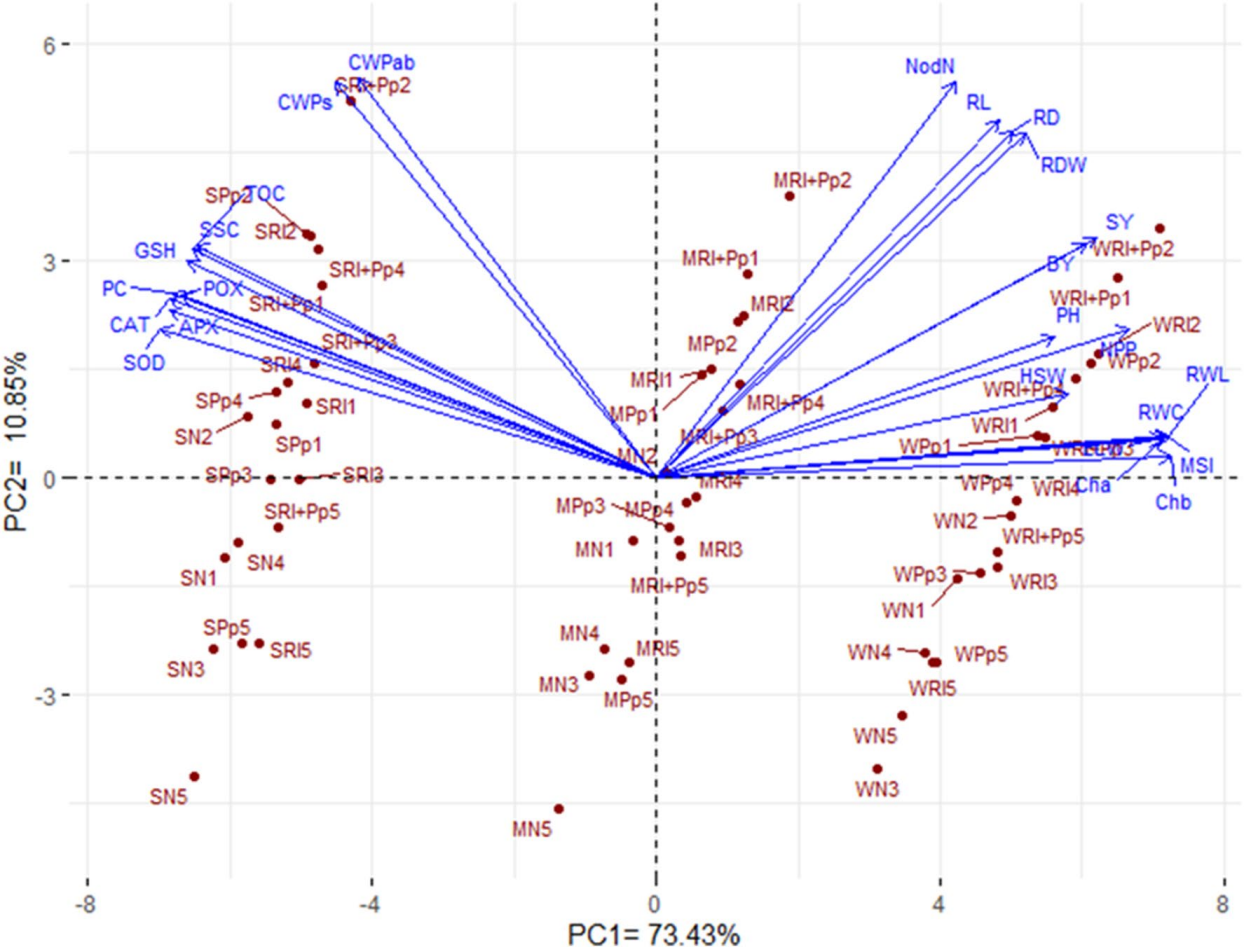
Biplot constructed from the first two principal components presenting the relationship among the evaluated agro-physiological and biochemical parameters affected by inoculation with *Rhizobium leguminosarum* (Rl) and *Pseudomonas putida* (Pp) versus non-inoculation (N) in five faba bean cultivars (1: Giza-716, 2: Giza-843, 3: Nubaria-3, 4: Sakha-4, and 5: Wadi-1) under three irrigation regimes (*W* well-watered, *M* moderate drought, *S* severe drought). *Chl a* chlorophyll *a*, *Chl b* chlorophyll *b*, *PC* proline content, *SSC* soluble sugar content, *RWC* relative water content, *MSI* membrane stability index, *ELWR* excised leaf water retention, *RWL* relative water loss, *CAT* catalase, *POX* peroxidase, *SOD* superoxide dismutase, *APX* ascorbate peroxidase, *GSH* glutathione, *TOC* α-tocopherol, *RL* root length, *RDW* root dry weight, *RD* root diameter, *NodN* number of nodules, *PH* plant height, *NPP* number of pods per plant, *HSW* 100-seed weight, *SY* seed yield, *BY* aboveground biomass, *CWP*_*ab*_ crop water productivity for aboveground biomass, *CWP*_*s*_ crop water productivity for seed yield.

Principal component analysis (PCA) was performed to investigate the association among the evaluated parameters and treatments. The first two principal components reflected the highest variability, approximately 84.28% (73.43% by PC1 and 10.85% by PC2) (Fig. 6). PCA1 corresponded with increasing the irrigation regimes from 50 to 100% ETc. Regardless of the inoculations and genotypes, well-watered conditions (100% ETc, W) were located on the positive side of PCA1, whereas severe drought conditions (50% ETc, S) were located on the negative side and moderate drought conditions (75% ETc, M) were located in the middle.

On the other hand, PCA2 corresponded to the dispersal of inoculation treatments from the bottom with the non-inoculated (N) to the top with the inoculation with both strains (Rl and Pp). Furthermore, the genotypes were spread out with different multi-dimensional spaces on PCA2. The Giza-716 (1) and Giza-843 (2) genotypes were mainly located on the top under well-watered and moderate drought conditions, whereas Giza-843 and Sakha-4 (4) were located on the top under severe drought conditions. In contrast, Wadi-1 (5) was located at the bottom under both severe and moderate drought conditions.

The traits were represented by parallel or close vectors, indicating a highly positive relationship, whereas those located approximately opposite (at 180°) signified a negative relationship. The 25 evaluated parameters could be classified into two groups. The first group (15 parameters) included agronomic traits, root parameters, plant–water parameters, and photosynthetic pigments. The second group (10 parameters) included enzymatic and non-enzymatic antioxidants and CWP_s_ and CWP_ab_.

## Discussion

The legume cultivars have high yield but are extremely sensitive to drought stress. Therefore, determining approaches for boosting legume growth and production under water-deficient conditions is crucial, particularly in arid environments. In this study, five *Vicia faba* cultivars were inoculated with *R. leguminosarum* and/or *P. putida* under three irrigation regimes and were investigated at the physiological, biochemical, and agronomic levels. Morris et al.^24^ and Huang et al. ^25^ revealed that simultaneous inoculation with distinct microbial isolates improves plant performance. Therefore, in the current study, two microbial inoculants were applied. *P. putida* produces phytohormones, secondary metabolites, bioactive compounds, and signaling molecules, which considerably boost metabolic efficiency and alleviate the adverse effects of drought stress^18,19^. *R. leguminosarum* efficiently produces natural N, which is an alternative to expensive inorganic N, and alleviates environmental pollution caused by the excessive application of inorganic N^26^. Symbiotic N_2_ fixation is essential, particularly in regions with newly reclaimed poor-fertility sandy soil, such as in the present study.

Photosynthetic pigments are vital predictors of plant health under environmental stresses^27^. Our results showed that moderate and severe drought stress caused a gradual but considerable decrease in the contents of photosynthetic pigments: Chl *a* and Chl *b*. Reduction in photosynthetic attributes is a major early plant response to water deficit, which substantially diminishes metabolite accumulation and plant productivity^28,29^. Previous studies revealed a positive relationship between chlorophyll content and yield traits of faba bean^30,31^. Enhancing the expression of photosynthetic pigments is imperative to improve the yield traits of faba bean under drought stress. The results showed that the application of *R. leguminosarum* and *P. putida* intrinsically enhanced the biosynthesis of photosynthetic pigments under moderate and severe drought stress conditions compared with that in non-inoculated plants. This may have resulted from an increased absorption efficiency of water and nutrients, which are important for the biosynthesis of photosynthetic pigments^32,33^. Moreover, PGPR application could affect plant physiological processes and the cell wall structure, which could enhance the synthesis of proteins and enzymes associated with pigment biosynthesis^34,35^.

Plant cellular osmolytes are important determinants of plant response to abiotic stresses^36,37^. Inoculation with *R. leguminosarum* and *P. putida* was associated with increased proline and soluble sugar contents in inoculated plants compared with those in non-inoculated plants. These osmoprotectants alleviated oxidative stress and boosted plant tolerance through diminishing ROS detoxification induced by drought stress, which conserved metabolic functions and enhanced physiological activities^38,39^. Furthermore, increased osmolyte accumulation reduced the cellular osmotic potential, which provided adequate water absorption from the soil, which in turn boosted cell turgor pressure, maintained the cell water status, and preserved the membranes even under severe drought stress^40,41^.

RWC, MSI, RWL, and ELWR are important indicators of plant–water relations under drought stress^42,43^. Under drought stress, *R. leguminosarum* and *P. putida* maintained plant RWC, MSI, ELWR, and RWL by boosting osmotic potentials and maintaining cellular membrane integrity and permeability. These results are consistent with those of previous studies on the potential of PGPR in enhancing plant–water relations and improving drought tolerance^44–46^.

ROS, including O_2_^−^, OH^−^, H_2_O_2_, and O_2_, are generated under water-deficit conditions and cause oxidative damage, decrease lipid peroxidation, and impair cell functions^36,45^. To prevent ROS accumulation and mitigate oxidative damage, plants develop enzymatic and non-enzymatic antioxidant defense systems^47,48^. Increasing the generation of ROS scavengers in stressed plants is a crucial intervention for mitigating negative drought impacts^49,50^. CAT reduces H_2_O_2_ levels and hinders the generation of OH^−^ radicals^51,52^. POX plays a vital role in eliminating H_2_O_2_ and reducing oxidative damage^53,54^. In the current study, the activities of POX, CAT, SOD, APX, GSH, and α-Toc substantially increased with increasing levels of water stress in all the cultivars. However, the activities of the enzymes significantly increased in the plants inoculated with *R. leguminosarum* and/or *P. putida* under drought conditions compared with those in the non-inoculated plants.

SOD is considered the first line of defense against ROS, and α-Toc plays a crucial role in the antioxidant system and in alleviating oxidative stress^55,56^. Inoculation with both isolates resulted in relatively superior values and substantially enhanced the enzyme activities in stressed plants compared with those in non-inoculated stressed plants. These results provide evidence for the beneficial effect of inoculation with PGPR in improving drought tolerance in faba bean plants by adjusting the activities of antioxidants and detoxifying ROS under drought conditions^57,58^.

At the proteome level, a change in the inoculated cultivars was observed in response to drought stress. A new positive band (4 kDa) was detected in Giza-843 inoculated with *R. leguminosarum* and *P. putida* under severe drought stress but was absent under well-watered conditions (Table 5). This finding suggests that the protein is translated, and its level was increased to cope with drought stress. Recently, Rashid et al.^59^ reported similar proteome changes in plants inoculated with PGPR and cultivated under drought stress. For instance, new proteins were expressed in response to drought stress, including adenosine triphosphate (ATP) synthase. This suggests that the expression of ATP synthase is highly induced in response to drought stress. Furthermore, Lim and Kim^60^ demonstrated that unique proteins were differentially expressed in plants inoculated with PGPR under drought stress compared with that in non-inoculated plants. They reported an association between the detected proteins (S-adenosylmethionine synthetase, dehydrin-like protein, vacuolar H-ATPase and adenosine kinase) and enhanced drought tolerance.

The root parameters (root length, DW, root diameter, and number of nodules) significantly decreased in plants exposed to moderate and severe drought conditions compared with those in non-stressed plants. Inoculated plants demonstrated improved root parameters compared with those in non-inoculated plants, particularly under drought stress. PGPR application, especially the combination of two strains, can enhance the uptake of mineral nutrients, which consequently provides balanced nutrition^61,62^.

The availability of mineral nutrients can increase the size and number of nodules, elevate the assimilated amount of N, and improve bacterial density in the soil surrounding the root^63,64^. Furthermore, root mass and length expansion provided more effective sites for nodulation^25,41,65^. In addition, nodule formation and root growth were enhanced by the secretion of phytohormones (e.g., cytokinins, auxins, and gibberellins), ammonia, exopolysaccharides, nitrogenase activity, and ACC deaminase^66,67^. Studies have shown that co-inoculation with different PGPRs enhanced early nodule initiation and root development in various crops. Knezevic-vukcevic^68^ demonstrated that co-inoculation of common bean with *Rhizobium* and *Bacillus* strains increased the number of nodules compared with that with inoculation with *Rhizobium* alone. Korir et al.^62^ reported that co-inoculation of common bean plants with *Paenibacillus polymyxa* and *Bacillus megaterium* considerably increased root DW compared with inoculation with *Rhizobium* alone or with non-inoculation treatment.

The agronomic traits were increased due to promotional effects in photosynthetic pigments, proline content, soluble sugar content, RWC, MSI, ELWR, RWL, and enzymatic and nonenzymatic antioxidants. The combination of both strains increased the values of all agronomic traits compared with those in non-inoculated plants, particularly under drought stress. Several studies have reported that the combination of *Rhizobium* with *Bacillus* or *Pseudomonas* displayed great potential to increase crop yields under stress conditions^32,69,70^. Khan et al.^41^ showed that inoculating chickpea seeds with *Bacillus thuringiensis*, *Bacillus subtilis*, and *Bacillus megaterium* enhanced the nodule number, plant height, seed weight, seed yield, and total biomass, which were higher in treated chickpea plants cultivated in poor-fertility sandy soil compared with the non-inoculated plants. Alkowni et al.^71^ showed that inoculation with *Pseudomonas putida* considerably increased the biomass and yield of barley, pearl millet, and clover under saline stress.

Faba bean is a legume crop that is sensitive to water stress; hence, identifying drought-tolerant genotypes is crucial to attenuate the devastating impacts associated with drought stress in arid environments. Agro-physiological and biochemical attributes were employed to evaluate the response of five faba bean cultivars to water deficit. The Giza-843 cultivar displayed the highest physiological traits, root parameters, agronomic traits, CWP_g,_ and CWP_b_, followed by the Giza-716 cultivar, under moderate drought, whereas Giza-843 had the highest physiological traits, root parameters, agronomic traits, CWP_g,_ and CWP_b_, followed by Sakha-4, under severe drought conditions. These cultivars tolerated water stress by boosting the efficacy of photosynthesis, plant–water relations, osmotic adjustment, and enzymatic antioxidant activities. Therefore, cultivating these drought‐tolerant cultivars is a preferred approach to ameliorate CWP and improve seed and biological yields, mainly in arid environments. The Wadi-1 cultivar showed the lowest values of these parameters under drought stress. Similarly, Siddiqui et al.^72^ and Mansour et al.^7^ reported significant differences among faba bean genotypes under drought stress. These studies demonstrated that drought stress-tolerant genotypes exhibited high photosynthetic pigment contents, enhanced plant–water relations, and increased enzyme activities, which were related to high seed yield compared with that of sensitive genotypes.

At the protein level, two new positive protein bands with molecular weights of 55 and 27 kDa were detected in non-inoculated Giza-843 plants under the drought stress and well-watered conditions (Table 5). These new bands indicated the activity of this cultivar under drought stress. In contrast, no difference in the protein banding pattern was observed between non-inoculated Wadi-1 plants under well-watered conditions and those under drought conditions, confirming the sensitivity of this cultivar to drought stress. Furthermore, the band intensity increased at molecular weights of 17, 30, and 50 kDa in Wadi-1 compared with that in Giza-843, whereas the band intensity increased at molecular weights of 22 and 40 kDa in Giza-843. The increase in band intensity and other results may be a part of the metabolic alterations in response to severe drought conditions in addition to genetic background interactions. Similarly, Wang et al.^73^ identified responsive protein bands that were associated with drought tolerance at the proteome level. They reported that these proteins were exclusively expressed under drought stress and played an important role in biosynthesis, photosynthesis, and energy metabolism to cope with water-deficit conditions.

Thus, moderate and severe drought stress gradually and significantly reduced faba bean growth and productivity. However, the application of PGPR, particularly the combination of *R. leguminosarum* and *P. putida*, improved all the evaluated physio-biochemical attributes and increased seed yield and CWP. The cultivars Giza-843 and Sakha-4 showed the highest tolerance under severe drought stress. These tolerant cultivars can be inoculated with both strains to decrease losses in crop yield in faba bean plants cultivated in poor soils and in arid environments.

## Conclusion

PGPR application, particularly co-inoculation with *R. leguminosarum* and *P. putida*, attenuated the detrimental effects of drought stress and ameliorated seed yield and CWP by enhancing the expression of photosynthetic pigments, plant–water relations, root parameters, and the components of the enzymatic and non-enzymatic defense system. The evaluated cultivars showed substantial genetic differences under drought stress: Giza-843, Giza-716, and Sakha-4 were more tolerant under drought stress. In contrast, Wadi-1 is a drought-sensitive cultivar based on physio-biochemical and agronomic parameters. The biochemical markers efficiently reflected the genetic diversity among faba bean genotypes inoculated with PGPR under different irrigation regimes.

## Materials and methods

### Experimental site and agricultural treatments

A field experiment was conducted during the 2018–2019 and 2019–2020 growing seasons at Elkhatara experimental farm, Alsharqia, Egypt (30° 36′ N, 31° 46′ E). This region is dry, with low precipitation and an annual rainfall of approximately 60 mm (Table S1). The soil of the experimental site is classified as sandy (i.e., 95.16% sand, 3.45% silt, and 1.39% clay). In both growing seasons, faba bean plants were sowed during the optimum period, which was the first week of November. Marshal (carbosulfan 25% EC; 500 ml ha^−1^) and Ridomil Gold 480 SL (mefenoxam; 2.4 L ha^−1^) were applied for pest control and disease control, respectively. Phosphorus (P) and potassium (K) were added before sowing at a rate of 75 kg P_2_O_5_ ha^−1^ and 110 K_2_O ha^−1^, respectively. N fertilizer was applied once at sowing at a basal dose at a rate of 45 kg N/ha as ammonium sulfate (21% N). A single N dose was applied in the poor sandy soil of the evaluated arid region due to the low N-fixing capacity of faba bean plants at the initial growth stage to enhance plant growth and induce the growth of uniform plant stands.

### Plant material and irrigation regimes

Five commercially cultivated faba bean (*Vicia faba* L.) cultivars, i.e., Giza-716, Giza-843, Nubaria-3, Sakha-4, and Wadi-1, were used (Table S2). These cultivars are recommended as high-yielding commercial cultivars. These cultivars complied with international, national, and institutional guidelines and legislation.

The experimental design was a split–split plot with three replications. Irrigation levels were located in the main plots, whereas the cultivars and inoculations were randomized in sub-plots and sub-sub-plots, respectively. Each plot comprised six 5-m long rows with a 0.70-m space between rows and a 0.15-m space between plants, resulting in an average plant population of 190,475 plants ha^−1^. Each hill was sown with four seeds and thinned to two seedlings directly after complete germination.

The cultivars were assessed under three water irrigation regimes based on ETc replacement following the crop coefficient approach^74^. Daily reference evapotranspiration was estimated from weather data employing the FAO-56 standardized Penman–Monteith equation, as described by Allen et al.^74^. During the first and second growing seasons, the cumulative amount of full irrigation regime (100% ETc) was 400 and 412 mm ha^−1^, respectively. The amount of full irrigation decreased by 25% and 50% after applying the moderate and severe drought stress conditions. During the two growing seasons, the moderate drought condition was 300 and 309 mm ha^−1^, respectively, and the severe drought condition was 200 and 206 mm ha^−1^, respectively. The drip irrigation system was employed using drip laterals with a 0.7-m space between them and an emitter spacing of 0.30 m. The irrigation sectors had valves and pressure gauges to maintain the operating pressure at 1 bar and emitter flow rate at 4 Lh^−1^. A flow meter was employed to determine the targeted amount of irrigation water for each irrigation regime. Drought conditions were applied from seedling establishment to physiological maturity. Two weeks before harvesting (mid-April), irrigation was completed in both growing seasons.

### Strains used and inoculation preparation

Two PGPR strains, namely, *R. leguminosarum* biovar *viciae* (USDA 2435) and *P. putida* (RA MTCC5279), were obtained from the Agricultural Microbiology Department, Zagazig University. These strains were selected based on their growth-promoting traits under drought stress from a previous preliminary greenhouse experiment (unpublished data).

For preparing bacterial suspensions, the strains were streaked on plate count agar (Oxoid, CM0325, Basingstoke, Hampshire, England) from glycerol stocks stored at − 80 °C and incubated for 72 h at 28 °C. Single colony from the plate count agar was transferred to a tube containing 10 mL of tryptic soy broth (TSB) (Oxoid, CM0129) for *Pseudomonas* and yeast extract mannitol (YEM) medium for *Rhizobium*^75^. Overnight cultures were obtained by incubating the broth for 48 h at 30 °C for the *Pseudomonas* (8.5 log CFU/mL) and *Rhizobium* (9.1 log CFU/mL) strains.

The inocula for field inoculation were prepared in flasks using YEM and TSB containing *Rhizobium* and *Pseudomonas*, respectively, without agar, according to the method described by Ahmad, et al.^76^. YEM media were prepared using a standard composition (0.5 g of yeast extract, 15 g of mannitol, 0.5 g of K_2_HPO_4_, 0.2 g of MgSO_4_.7H_2_O, and 0.1 g of NaCl, with the pH adjusted to 7.0), whereas TSB was prepared using a standard composition as outlined by the manufacturer. Each flask containing broth was inoculated with the corresponding *Rhizobium* and *Pseudomonas* strains and incubated at 28 °C for 72 h under shaking (100 rpm) conditions.

The optical density (OD) of each strain was determined at 540 nm, and the population was confirmed by plating in suitable media. The OD_540_ of the two strains (8–9 log CFU/mL) was attained by dilution with Ringer’s solution before seed inoculation. The cell density was measured using the dilution plate procedure. Briefly, the agar plates were inoculated with 1 mL of each diluted strain. The cell density was determined after 48 h of incubation and varied between 8 and 9 log CFU/mL. The seeds were placed in the *P. putida* suspension for 12 h. Inoculation with *R. leguminosarum* was applied before sowing at a rate of 10 g/kg seeds under shading using sucrose solution (20%) as an adhesive agent for seed coating.

### Measurement of physio-biochemical parameters

Five faba bean plants from each plot were collected 50 days after sowing. The leaves on the fifth node of the main stem were used for determining the physiological and chemical parameters. The photosynthetic pigments (Chl *a* and Chl *b*) were extracted from fresh leaves (0.1 g) using pure acetone following Fadeel’s method^77^.

The pigments were filtered, and the OD of the filtrate was determined spectrophotochemically at 662 and 644 nm for Chl *a* and Chl *b*, respectively. The contents of the pigments [mg/g fresh weight (FW)] were determined using the formula described by Vonwettstein^78^. RWC was determined according to the method of Barrs and Weatherley^79^. FW was recorded from the leaves, which were then soaked in water for 3 h, and the turgid weight (TW) was calculated. Then, the samples were dried in the oven at 80 °C for 24 h, and DW was recorded. The RWC was estimated as follows:

$${\text{RWC}}=\frac{{\text{FW}} - {\text{DW}}}{{\text{TW}} -{\text{DW}}} \times 100$$

MSI was estimated using 200 mg of fresh material in test tubes containing 10 cm^3^ of double-distilled water in two sets. The first set was heated at 40 °C for 30 min in a water bath, and the electrical conductivity of the solution was measured on a conductivity bridge (C_1_). The second set was boiled at 100 °C for 10 min, and the electrical conductivity was measured on a conductivity bridge (C_2_). MSI was estimated as outlined by Rady^80^ using the following formula:

$${\text{MSI}}=\left\{1-\left(\frac{{\text{C1}}}{{\text{C2}}}\right)\right\}\times 100$$

Four new leaves were freshly weighed and then left for 4 h to wilt at 25 °C and reweighed (WW4h). ELWR was estimated using the following equation, as described by Farshadfar et al.^81^:

$${\text{ELWR }}\left(\%\right)=\left[1-\frac{{\text{FW}} -{\text{WW4h}}}{{\text{FW}}}\right]\times 100$$

For each treatment, four leaves were freshly weighed (FW), wilted for 4 h at 35 °C in an incubator, reweighed (WW4h), and oven-dried for 24 h at 72 °C to obtain DW. RWL was estimated using the following equation, as described by Gavuzzi et al.^82^:

$${\text{RWL}}(\%)=\frac{({\text{FW}} -{\text{WW4h}})}{({\text{FW}} - {\text{DW}})}\times 100$$

Proline accumulation in leaves was evaluated as outlined in the study by Bates et al.^83^.

A fresh leaf (0.1 g) sample was grounded with 10 mL of 3% (w/v) aqueous sulfosalicylic acid, and the homogenate was filtered using a Whatman grade 2 filter paper. Then, 1 mL of the filtrate was reacted with 1 mL of acid ninhydrin reagent and 1 mL of glacial acetic acid in a test tube for 1 h at 100 °C, and the reaction was terminated in an ice bath. Two milliliters of toluene was added to the mixture, and the absorbance of the upper toluene layer at 520 nm was determined using Shimadzu UV-2101/3101 PC scanning spectrophotometer (Shimadzu Corporation Analytical Instruments Division, Kyoto, Japan).

The total soluble sugar content was determined by washing 0.2 of g leaves with 5 mL of 70% ethanol and homogenizing them with 5 mL of 96% ethanol. The obtained extract was centrifuged at 3500×*g* for 10 min, and the supernatant was stored at 4 °C ^84^. Anthrone (3 mL) was added to 0.1 mL of supernatant. The mixture was incubated in a hot water bath for 10 min. The absorbance was measured at 625 nm using UV-2101/3101 PC scanning spectrophotometer (Shimadzu).

The levels of GSH (µmoL g^−1^ of leaf FW) were determined following the method described by Griffith^85^. The procedures described by Konings et al.^86^ and Ching and Mohamed^87^ were used to measure α-Toc levels using a high-performance liquid chromatography system (HPLC; SpectraLab Scientific Inc., ON, Canada) with methanol:water in a 94:6 (v/v) ratio as the mobile phase with a flow rate of 1.5 mL/min, and a UV detector, which was set at 292 nm.

The extract was prepared following the method described by Vitória, et al.^88^. CAT levels were measured as described by Chance and Maehly^89^. The method of Thomas et al.^90^ was used to assess POX activity. APX levels were measured as outlined by Fielding and Hall^91^. SOD activity was evaluated by scoring the decrease in absorbance of superoxide-nitro blue tetrazolium complex by the enzyme^92^.

### Measurement of root parameters

Five plants were harvested from each plot 50 days after sowing. Bulk soil was separated by gently shaking the roots and then washed using tap water. The shoots were carefully separated above the collar region. The nodules were counted on the main and lateral roots. Taproot length (cm) was measured from the collar region to the tip of the main root. Root diameter (cm) was measured at the collar region. The roots were dried in the oven at 70 °C for 48 h and then weighed.

### Growth and yield parameter measurements

At physiological maturity (when > 80% of the pods and stems lost their green pigmentation and turned black), plant height (cm) was recorded from the ground to the top of the plant from readings of 10 plants in each plot. Similarly, the number of pods plant^−1^ was estimated.

Seed weight was obtained from the weight of four sets of 100 seeds. Seed yield and aboveground biomass were estimated by harvesting three rows for a total area of 10.5 m^2^/plot and converted into kg ha^−1^.

### CWP

CWP (kg m^−3^) was estimated as the ratio of seed yield or biological yield (kg ha^−1^) to ET (mm) following the method described by Fernández et al.^93^ using the following formulas:

$${\text{CWP (kg m}}^{-3})=\frac{{\text{Yield}} }{{\text{ET}}}$$

$${\text{ET}}= {I}+P+{C}+{D}\pm {R }\pm \Delta {S}$$

where I is the irrigation amount (mm), P is seasonal precipitation (mm), C is the capillary rise to the root zone (mm), D is deep percolation (mm), R is the surface runoff (mm), and ∆S is the soil moisture variation in the crop root zone (mm). C was neglected as the groundwater table is 15 m below the ground surface. D and R were considered negligible because a drip irrigation system was employed. Soil water content was determined using the oven-drying procedure for all experimental plots.

### Electrophoresis of seed storage protein using SDS–PAGE

The protein banding pattern was assessed using the SDS–PAGE technique. The most tolerant and sensitive cultivars were selected based on their physio-biochemical, morphological, and agronomic traits at the end of the experiment. The seeds of these cultivars were collected from well-watered (100% ETc) and severe stress (50% ETc) treatments. Total seed storage protein was extracted and assessed for the cultivars under drought stress and well-watered conditions, and the protein expression patterns were compared^94^.

Then, 100 µg of the protein from different treatments were collected and combined with 10 mL of sample buffer in a microfuge tube, boiled for 4 min, and incubated at 48 °C for 30 min. Thereafter, the samples containing equal amounts of proteins were inserted into the wells of polyacrylamide gels (Sigma–Aldrich Chemie GmbH, Taufkirchen, Germany). The medium-range-molecular-weight markers (Bangalore Genei, India) were applied, and electrophoresis was performed at a constant voltage of 75 V for 2 h.

### Statistical analysis

Analysis of variance (ANOVA) was performed for the split–split plot design in three replications across two growing seasons. Combined ANOVA was performed to analyze the differences among the irrigation regimes, cultivars, inoculations, and their interactions across the two growing seasons using Bartlett’s test for homogeneity of variances.

The combined analysis revealed homogenous variances across the two growing seasons for the measured traits; consequently, the data of the two growing seasons were combined. The differences among the evaluated treatments were discriminated using the protected least significant difference at a significance level of *p* ≤ 0.05.

All statistical analyses were performed using R version 4.1.1 (R Foundation for Statistical Computing, Vienna, Austria).

## Supplementary Information

Supplementary Information.

## Data Availability

The data presented in this study are available upon request from the corresponding author.

## Abbreviations

PGPR: Plant growth-promoting rhizobacteria
ETc: Crop evapotranspiration
Chl *a*: Chlorophyll *a*
Chl *b*: Chlorophyll *b*
FW: Fresh weight
DW: Dry weight
RWC: Relative water content
MSI: Membrane stability index
ELWR: Excised leaf water retention
RWL: Relative water loss
CAT: Catalase
POX: Peroxidase
SOD: Superoxide dismutase
APX: Ascorbate peroxidase
GSH: Glutathione
α-Toc: α-Tocopherol
ROS: Reactive oxygen species
SDS–PAGE: Sodium dodecyl sulfate–polyacrylamide gel electrophoresis
CWP_s_: Crop water productivity for seed yield
CWP_ab_: Crop water productivity for aboveground biomass
PCA: Principal component analysis
ACC: 1-Aminocyclopropane-1-carboxylic acid
TSB: Tryptic soy broth
YEM: Yeast extract mannitol
