# Supplementary material for: Enhancement of drought tolerance in diverse Vicia faba cultivars by inoculation with plant growth-promoting rhizobacteria under newly reclaimed soil conditions
Source: Sci Rep. 2021 Dec 17;11:24142. doi: 10.1038/s41598-021-02847-2 (PMC8683512; doi:10.1038/s41598-021-02847-2)
Supplement: Supplementary file 1 — Supplementary Information. [file 41598_2021_2847_MOESM1_ESM.pdf]

Supplementary Data

Enhancement of drought tolerance in diverse *Vicia faba* cultivars by inoculation with plant growth-promoting rhizobacteria under newly reclaimed soil conditions

Elsayed Mansour, Hany A.M. Mahgoub, Samir A. Mahgoub, El-Sayed E.A. El-Sobky, Mohamed I. Abdul-Hamid, Mohamed M. Kamara, Synan F. AbuQamar[*], Khaled A. El-Tarabily[*], El-Sayed M. Desoky

*** Correspondence:**
Prof. Synan AbuQamar: sabuqamar@uaeu.ac.ae
Prof. Khaled El-Tarabily: ktarabily@uaeu.ac.ae

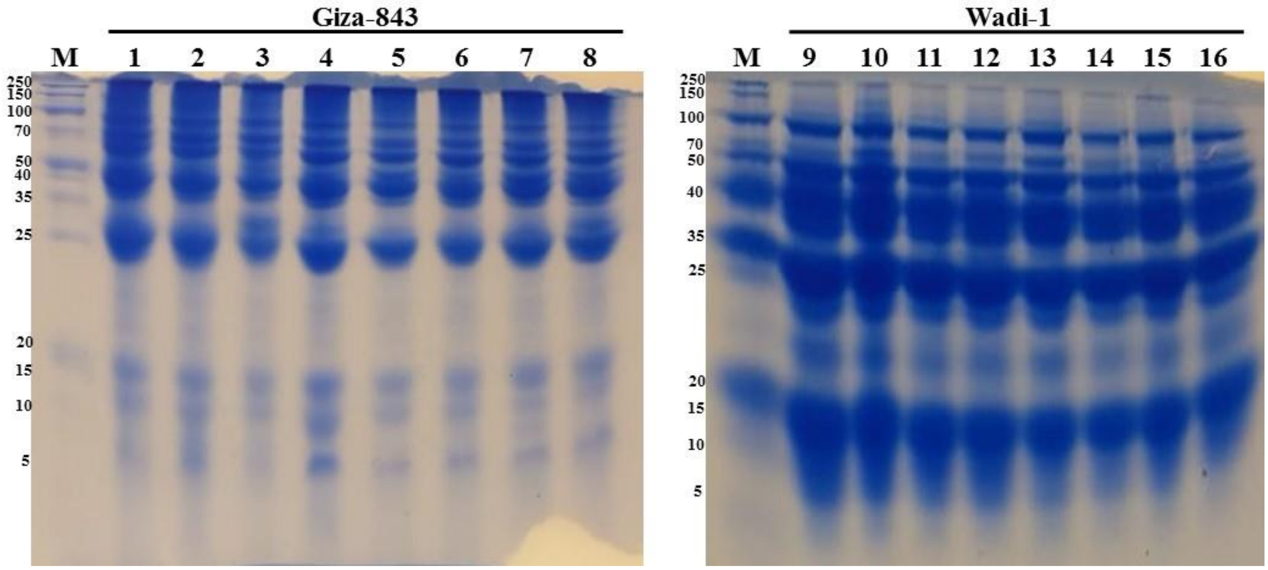

Fig. S1. SDS-PAGE of seed protein banding patterns of two faba bean cultivars, Giza-843 (left) and Wadi-1 (right) inoculated with *Rhizobium leguminosarum* and/or *Pseudomonas putida* under well-watered and severe drought stress separated on 15 % SDS-PAGE. Bands (1-8) belong to Giza-843 cultivar, while (9-16) belong to Wadi-1. M, protein marker; 1 and 9, non-inoculated plants under well-watered conditions; 2 and 10, *P. putida*-inoculated plants under well-watered; 3 and 11, *R. leguminosarum*- inoculated plants under well-watered conditions; 4 and 12, inoculated-plants with *R. leguminosarum* and *P. putida* under well-watered conditions; 5 and 13, non-inoculated plants under severe drought stress conditions; 6 and 14, *P. putida*-inoculated plants under severe drought; 7 and 15, *R. leguminosarum*- inoculated plants under severe drought stress conditions; 8 and 16, inoculated-plants with *R. leguminosarum* and *P. putida* under severe drought stress conditions.

Table S1. Monthly minimum (Tmin, ◦C) and maximum (Tmax, ◦C) temperatures and precipitation (Prec., mm).

Month	2018-2019			2019-2020			35-year average (1986-2020)		
	Tmin	Tmax	Prec.	Tmin	Tmax	Prec.	Tmin	Tmax	Prec.
November	14.4	26.9	5.7	14.9	28.5	5.9	13.6	25.6	9.7
December	10.9	21.2	9.8	9.6	21.0	9.2	9.8	21.1	7.1
January	7.0	19.1	10.3	7.3	18.0	9.6	8.2	19.5	11.0
February	9.0	20.1	12.9	8.1	20.5	15.1	9.1	20.7	10.3
March	11.3	23.0	11.2	10.1	24.8	15.6	11.3	23.7	8.1
April	13.8	26.2	3.2	12.4	25.3	4.1	14.2	28.0	4.8

.

Table S2. Name, pedigree and origin of the used faba bean cultivars.

**Cultivar**	**Pedigree**	**Origin**
Giza-716	83/453/503 × 83/824/461	Egypt
Giza-843	461/845/83 × 561/2076/85	Egypt
Nubaria-3	Selection in Ahnasiaz	Egypt
Sakha-4	Sakha-1 × Giza-3	Egypt
Wadi-1	Giza Blanka × Trible-White	Egypt
